# Does overreaching from endurance-based training impair sleep: A systematic review and meta-analysis

**DOI:** 10.1371/journal.pone.0303748

**Published:** 2024-05-29

**Authors:** Conor Murphy, Steinunn Anna Svansdottir, Olivier Dupuy, Julien Louis

**Affiliations:** 1 Physical Activity, Physical Education, Sport and Health Research Centre (PAPESH), Sports Science Department, School of Social Sciences, Reykjavik University, Reykjavik, Iceland; 2 Laboratory MOVE (UR 20296), Faculty of Sport Sciences, University of Poitiers, Poitiers, France; 3 Research Institute for Sport and Exercise Sciences (RISES), Liverpool John Moores University, Liverpool, United Kingdom; Portugal Football School, Portuguese Football Federation, PORTUGAL

## Abstract

**Background:**

Overreaching is often linked to a deterioration in sleep quality, yet a comprehensive review is lacking. The aim of this systemic review and meta-analysis was to synthesise the literature and quantify the effect of overreaching from endurance-based training on sleep quality.

**Method:**

The review was conducted following the PRISMA guidelines. The final search was conducted in May 2023 using four electronic databases (Web of Science Core Collection, MEDLINE, Cochrane Central Database, SPORTDiscus). Studies were included for a qualitative review, while random-effects meta-analyses were conducted for objective and subjective sleep.

**Results and discussion:**

The search returned 805 articles. Fourteen studies were included in the systematic review; Three and eight articles were eligible for the meta-analyses (objective and subjective, respectively). On average, the overreaching protocols were sixteen days in length (6 to 28 days) and included exercise modalities such as cycling (number of studies [k] = 5), rowing (k = 4), triathlon (k = 3), running (k = 2), and swimming (k = 1). Actigraphy was the only form of objective sleep measurement used across all studies (k = 3), while various instruments were used to capture subjective sleep quality (k = 13). When comparing objective sleep quality following the overreaching intervention to baseline (or a control), there was a significant reduction in sleep efficiency (mean difference = -2.0%; 95% CI -3.2, -0.8%; Glass’ Δ = -0.83; p < 0.01). In contrast, when comparing subjective sleep quality following the overreaching intervention to baseline (or a control), there was no effect on subjective sleep quality (Glass’ Δ = -0.27; 95% CI -0.79, 0.25; p = 0.08). Importantly, none of the included studies were judged to have a low risk of bias. While acknowledging the need for more high-quality studies, it appears that overreaching from endurance-based training can deteriorate objective sleep without influencing the perception of sleep quality.

**Protocol registration:**

This protocol was registered in The International Prospective Register of Systematic Reviews (PROSPERO) on 21^st^ November 2022, with the registration number CRD42022373204.

## Introduction

Consistent and methodological training often underpins the success of elite endurance athletes. Yet while training philosophies may differ in terms of training volume and intensity distributions [1], the prescribed training requires a minimum effective stimulus to elicit physiological adaptations. Fatigue and recovery play an important balancing role in garnering either positive or negative responses to exercise [2]. Following excessive training, the loss of balance between recovery and fatigue can lead to a short-term decrement in physical performance more commonly known as functional overreaching [3, 4]. While functional overreaching causes a decrease in performance, appropriate recovery can result in improved performance compared with baseline levels representing a “supercompensation” effect [3]. Nonetheless, this fatigue must be managed through comprehensive recovery; otherwise, functional overreaching may soon turn into non-functional [3]. With the latter, the body fails to fully recover causing further maladaptation of performance and displaying symptoms of physiological and psychological distress. The progression of non-functional overreaching can also manifest in overtraining syndrome, which is associated with a long-term decrement in performance and dysregulation of several biological, hormonal, and psychological processes [3].

Sleep is recognised as an integral part of exercise recovery. Sleep is involved in the maintenance of physiological systems within the body via its links with immune function [5], hormone regulation [6, 7], and metabolic efficiency [8]. Furthermore, the positive impact of sleep on the psychological system, including plastic remodelling and alteration of mood states, is a much agreed-upon outcome of the sleep process [9, 10]. From a practical perspective, sleep loss studies highlight the ability of disturbed sleep to challenge exercise performance [11]. Despite the suggestion that sleep is fundamental for the recovery of athletes, increased training load is often considered a risk factor for deteriorations in sleep quality [12], while sleep itself has recently been posed as an indicator to detect overreaching [13]. For example, an expert consensus statement published in 2020 [14] suggested that overreaching and overtraining were associated with a decline in sleep quality reiterating the conclusions of an earlier opinion article in this area [13]. While both articles are informative, future research that systematically reviews the literature and explicitly defines the eligible studies’ inclusion and exclusion criteria is required. Furthermore, a detailed breakdown of each study’s design, characteristics, methods, measurements, and biases would indicate the quality of evidence in this area and highlight areas for future research.

The articles by Walsh et al. (2020) [14] and Lastella et al. (2018) [13] chose a generalised approach by evaluating all sports literature related to overreaching and sleep. Unfortunately, confounding factors that independently influence sleep, irrespective of overreaching, may differ between sports, making the true influence of overreaching on sleep challenging to discern. For example, how sleep disturbances transpire may differ depending on whether a sport includes characteristics such as contact and collisions, high cardiac loading, cognitive demands, or extensive muscle damage. Therefore, investigating the effect of overreaching on sleep might be more informative when reviewed on a sports classification basis. Due to their comparative simplicity, endurance sports may represent a feasible sports classification to assess the effect of overreaching on sleep. Endurance events are typically viewed as linear-based, thus, training load is more easily quantified when compared to other sports that are intermittent in nature, include significant technical components, or involve physical contact. From a research context, this represents an important feature as overreaching in experimental conditions is typically induced by abruptly increasing training load. Thus, endurance-based investigations might garner more confidence that changes in sleep quality are in fact a consequence of overreaching due to the better control of training load and other sports-specific confounding factors.

Therefore, the objective of this article was to conduct a systematic review and meta-analysis 1) describing the studies that have experimentally investigated the impact of endurance-based overreaching on subjective and objective sleep quality outcomes when compared to habitual training (within or between participants); 2) evaluating the risk of bias of all eligible studies; 3) quantitively assessing the effect of endurance-based overreaching on subjective and objective sleep quality; and 4) outlining practical applications and directions for future research.

## Methods

### Search strategy

Following the guidelines of the Preferred Reporting Items for Systematic Reviews and Meta-Analyses (PRISMA), a systematic search of the literature was conducted [15]. The search strategy included the use of four electronic databases (Web of Science Core Collection, MEDLINE, Cochrane Central Database, SPORTDiscus) that were systematically screened. The final search was completed on 12^th^ May 2023 with restrictions on language (English and French only), but none relating to the date range of studies. Along with keyword searching, Medical Subject Headings (MeSH) were used to search the aforementioned libraries. Search terms were characterised under three headers representing this review’s main constituents: (1) sleep; (2) overreaching; and (3) endurance exercise. Each header was combined within the search strategy by “AND” while search terms within each header were separated by “OR”. A comprehensive breakdown of the search strategy is provided in S1 Appendix. The protocol was registered in The International Prospective Register of Systematic Reviews (PROSPERO) on 21st November 2022, with the registration number CRD42022373204.

### Eligibility criteria

Population, Intervention, Comparison, Outcomes, and Study Design (PICOS) were considered when defining the limits of the review. In order to be eligible, studies were required to satisfy pre-defined inclusion criteria:

1. the study identified an increased training period (volume, intensity, or both);
2. the exercise modality was specific to linear-based endurance sports;
3. the participants (or a subset) were classified as overreached;
4. extractable sleep quality outcomes pre- and post-overreaching were reported in the full-text or supplementary files and;
5. the study was published in a peer-reviewed journal as a full-text article.

Exclusion criteria were also set to limit the inclusion of studies containing confounding factors. Studies were excluded if they: (1) had an inappropriate study design (i.e. retrospective or non-specific in nature); (2) were associated with sleep restriction; or (3) did not confirm overreaching using statistical methods.

Linear-based sports for the context of this review were considered as those relating to distance running, cycling, triathlon, swimming, skiing, canoeing, rowing, and kayaking. Overreaching was characterised by a decrement in performance from before to after the intervention (i.e., increased training). In the context of this review, objective sleep continuity variables (sleep latency, number of awakenings > 5 minutes, wake after sleep onset, sleep efficiency, sleep architecture) were indicators of sleep quality as previously recommended [16]. Subjective sleep quality was considered as any data that implied an increase or decrease in perceived sleep quality (answers reflecting sleep continuity or ratings of the sleep period). Although not as a measure of quality, total sleep time was reported if collected alongside sleep quality indicators. For the qualitative analysis, any studies where the comparative data in the full-text or supplementary files could be interpreted as an increase, no change, or a decrease in sleep quality were considered. However, a minimum of continuous raw (mean, standard deviation, sample size) pre- and post-sleep quality data must have been extractable from either the main text or supplementary file to be included in the meta-analyses. Overreached and control subgroups were included in the meta-analysis, but subgroups considered acutely fatigued were not. Sleep data from broader questionnaires were acceptable if they were subcategorised within the article.

### Study selection and data extraction

Following the execution of the search strategy, the results were collated and duplicates removed using the Mendeley Reference Management software (Elsevier, London, England). Two authors (CM + SS) screened the titles and abstracts of each article to determine their eligibility for full-text screening. Full texts then were reviewed in accordance with the predefined eligibility criteria. A custom data extraction sheet was used by CM to extract relevant data from the eligible studies. This sheet was devised with consideration to the Cochrane Consumers and Communication Review Group’s data extraction template [17]. Extracted data included: (1) study and participant characteristics (2) methodology (3) measurements and (4) outcomes. When relevant data were not reported or clarification was sought, the lead author was contacted. Authors related to eight studies [18–25] were contacted via email, two responded, and two provided the required data [23, 24]. If relevant data was presented in the study figures, they were extracted using Plot Digitizer (https://plotdigitizer.com/app).

### Risk of bias assessment

The risk of bias assessment was conducted independently by two authors (CM + JL) using the most recent versions of Cochranes tools for systematic reviews. In order to handle different study designs, the revised Risk of Bias for randomised trials (RoB2) tool was used to assess randomised control trials (RCT) [26], while the Risk of Bias In Non-randomised Studies of Interventions (ROBINS-I) tool was used to grade non-randomised trials [27]. Risk of bias for RCTs and non-RCTs were judged as “low, some concern or high” and “low, moderate, serious, critical, or not applicable”, respectively. Risk-of-bias VISualization tool was used to produce traffic light plots for each assessment [28].

### Meta-analyses

Raw sleep quality data (mean, standard deviation, sample size) were required for use in the meta-analyses. Mean standardised differences were chosen within the meta-analysis when the included studies employed the same measurement instruments. When the measurement instruments differed, standardised mean differences (Glass’ Δ) were used in order to permit between-study comparisons:

$$\Delta =\frac{mX1-mX2}{sdX2}$$ (1)

where mX1, mX2 and sdX2 are the post-intervention mean, the control group mean (or baseline mean for non-RCTs) and the control group standard deviation (or baseline standard deviation for non-RCTs). Control group comparisons were selected over baseline comparisons when both were available.

Two meta-analyses were conducted: 1) to determine the effect of overreaching on objective sleep quality and 2) to determine the effect of overreaching on subjective sleep quality. When a study used more than one method to assess either objective or subjective sleep quality then the most appropriate was chosen for the meta-analysis, see selections in Table 1. Subgroup analyses were performed with respect to study design (RCTs and non-RCTs). While a subgroup analysis was conducted to account for differences in study design, the meta-analyses were still performed using random effects models to account for other aspects of heterogeneity. Glass’ Δ were interpreted as follows; trivial = < 0.2, small effect = 0.2–0.49, moderate effect = 0.5–0.79, large effect = ≥ 0.8 [29]. Heterogeneity was explored using both the I^2^ index (≥ 75%) and Cochran’s Q test (p < 0.10) [30]. All meta-analyses were conducted using the “meta” R package (version 4.2.1).

**Table 1:** Overview of the reviewed studies.

Study	Original sample (n; gender; control group^a^)	Training status (description in manuscript;$\overset{\cdot }{V}O_{2\text{max}}$)	Study design (type; description; exercise modality; performance tests)	Sleep quality measurements	Objective sleep quality post overreaching^c^ decrease↓ increase↑ unchanged ↔	Subjective sleep quality post overreaching^c^ decrease↓ increase↑ unchanged ↔
Achten *et al*., (2004)^b^	16; 16 males and 0 females 0/16 controls	Endurance trained runners running >50 km/wk for the previous two months, have at least 5 yrs running experience, and have a personal best for 10 km below 40 min; 64.7 ± 2.6 ml.kg^-1^.min^-1^ (standard error)	Crossover; Easy + intensified training (high carbohydrates or control): 3 + 7 days Wash-out: 10 days Easy + intensified training (high carbohydrates or control): 3 + 7 days; (Load Δ: not calculatable; time spent in anaerobic training zones) Running; 8 km TT	Diary of sleep patterns (provides a measure of sleep quality and total sleep time)	n/a	↔ Perceived sleep quality (within) ↔ Total sleep time (within) Results reflect “high carbohydrate” and “control” groups combined (both groups were overreached)
Bellinger et al. (2020)	24; 16 males and 8 females 0/24 controls	Highly trained middle-distance runners (800-m and 1500-m) had a consistent training history of at least 2 yrs in these events, and were without major injury interruption for the previous 3 months. Male runners had personal best times for the 800-m and 1500-m races of 119.47.8 s (range = 108.3–133.4 s) and 238.0±16.8 s (225.2–279.1 s), respectively, whereas female runners had times of 135.0± 8.6 s (124.1–153.4 s) and 284.1±18.8 s (257.4–321.4 s), respectively; Male: 73.3±4.3 ml.kg^-1^.min^-1^ Females: 63.2±3.4 ml.kg^-1^.min^-1^	Uncontrolled before and after study; Normal training: 21 days Increased training: 21 days Taper: 7 days; (Load Δ: +28%, sRPE x duration) Running; Maximal GXT	10-point sleep quality log^d^	n/a	↓ Perceived sleep quality (within)
Costello et al. (2022)	23; 23 males and 0 females 11/23 controls	Well-trained competitive endurance cyclists that held at least a category 3 British Cycling licence. According to $\overset{\cdot }{V}O_{2\text{max}}$ classifications, three could be categorised as trained, four highly trained, and five professional; 61.8 ± 6.5 ml.kg^-1^.min^-1^	Randomised control trial; Habitual training: 7 days Intensified training: 14 days Taper: 14 days; (Load Δ: +116%, sRPE x duration) Cycling; Maximal GXT and 60 min TT	Karolinska sleep diary^d^ (many questions assessing sleep quality and length) RESTQ-Sport (provides a measure of sleep quality)	n/a	↔ Perceived sleep quality (diary) (no group x time interactions in any of the diary questions) ↔ Perceived sleep quality (RESTQ-Sport) (no group x time interactions)
Coutts et al. (2007)	16; 16 males and 0 females 8/16 controls	Well trained triathletes regularly competing in triathlons for at least 3 yrs, performing more than six triathlons per year and training a minimum of 10 h/week. Ten of the athletes competed at an international level for their respective age groups; Control: 52.8 ± 4.7 ml.kg^-1^.min^-1^ Overreached: 54.9 ± 5.6 ml.kg^-1^.min^-1^	Randomised control trial; Easy training: 21 days Intensified training: 28 days Taper: 14 days; (Load Δ: +190%, sRPE x duration) Triathlon; Maximal GXT and 3 km TT	RESTQ-Sport^d^ (provides a measure of sleep quality)	n/a	↔ Perceived sleep quality (no group x time interaction)
Hausswirth *et al*., (2014)	40; 27 males and 0 females; 9/27 controls (only data for final sample)	Well-trained triathletes that have been competing for ≥3 yrs, training ≥7 times per week with a training volume >10 h/week; Control: 59.5 ± 3.6 ml.kg^-1^.min^-1^, Acute fatigue: 58.5 ± 5.9 ml.kg^-1^.min^-1^, Functionally overreached: 63.0 ± 4.1 ml.kg^-1^.min^-1^	Randomised control trial; Normal training: 21 days Moderate training): 7 days Overload training: 21 days Taper: 14 days; (Load Δ: +36% h/week) Triathlon; Maximal GXT	Actigraphy monitor^e^ 7-point sleep quality log^d^	↔ Sleep efficiency (between) ↓ Sleep efficiency (within) (group x time interaction) ↔ Sleep latency (no group x time interactions) ↔ Total sleep time (between) ↓ Total sleep time (within) (group x time interaction)	↔ Perceived sleep quality (no group x time interactions)
Jeukendrup et al. (1992)^b^	8; 8 males and 0 females; 0/8 controls	Competitive cyclists who had been racing for at least 2 yrs; 71.1 ± 5.2 ml.kg^-1^.min^-1^	Uncontrolled before and after study; Moderate training: 14 days Heavy training:14 days; (Load Δ: +40%, h/week and +50, % time spent in anaerobic training zones) Cycling; Maximal GXT and 8.51 km TT	25 item overtraining questionnaire (included a question related to sleep latency)	n/a	↓Perceived sleep quality (within)
Jurimae et al. (2002)^b^	17; 17 males and 0 females; 0/17 controls	National level rowers (Estonian national team); No $\overset{\cdot }{V}O_{2\text{max}}$ data	Uncontrolled before and after study; Baseline training: 28 days Increased training: 6 days; (Load Δ: +100%, h/week assumed) Rowing; 2000 m TT	RESTQ-Sport (provides a measure of sleep quality)	n/a	↓ Perceived sleep quality (within)
Jurimae et al. (2004)	21; 21 males and 0 females 0/21 controls	National level rowers; No $\overset{\cdot }{V}O_{2\text{max}}$ data	Uncontrolled before and after study; Baseline training: 28 days Increased training: 6 days; (Load Δ: +100%, h/week assumed) Rowing; 2000 m TT	RESTQ-Sport^d^ (provides a measure of sleep quality)	n/a	↓ Perceived sleep quality (within)
Jurimae et al. (2004)^b^	14; 14 males and 0 females 0/14 controls	National level rowers; 60.4 ± 6.1 ml.kg^-1^.min^-1^	Uncontrolled before and after study; Baseline training: 28 days Increased training: 6 days; (Load Δ: +100%, h/week assumed) Rowing; 2000 m TT	7-point sleep quality log	n/a	↔ Perceived sleep quality (within)
Killer et al. (2017)	15; 15 males and 0 females; 0/17 controls	Highly-trained cyclists with a cycling history of at least 3 yrs, currently cycling at least three times per week for a minimum of 2 h/day and had a $\overset{\cdot }{V}O_{2\text{max}}$ of ≥65 ml.kg^-1^.min^-1^; 72.2 ± 4.9 ml.kg^-1^.min^-1^	Crossover; Baseline training: 14 days Intensified training (high carbohydrates or control): 9 days Washout: 10 days Intensified training (high carbohydrates or control): 9 days; (Load Δ: +153%, h/week and +146%, time spent in anaerobic training zones) Cycling; Maximal GXT	Actigraphy monitor^e^	↓ Sleep efficiency (within) ↔ Sleep latency (within) ↔ Total sleep time (within) Results reflect “high carbohydrate” and “control” groups combined (both groups were overreached)	n/a
Schaal et al. (2015)	11; 0 males and 11 females; 0/11 controls	International level synchronised swimmers (French national team); 62.1 ± 0.3 ml.kg^-1^.min^-1^	Crossover; Normal training: 7 days Intensified training (WBC or no WBC): 14 days Taper: 9 days Intensified training (WBC or no WBC): 14 days; (Load Δ: +27%, RPE x minutes) Swimming; Submaximal GXT and 400-m TT	Actigraphy monitor^e^ 7-point sleep quality log^d^	↓ Sleep efficiency (within) ↑ Sleep latency (within) ↓ Total sleep time (within) Results reflect “no WBC” group (only group that was overreached)	↔ Perceived sleep quality (within) Results reflect “no WBC” group (only group that was overreached)
Urhausen et al. (1998)^b^	23; 23 males and 0 females 0/23 controls	Male endurance athletes; 61.2 ± 7.5 ml.kg^-1^.min^-1^	Uncontrolled before and after study; 5 periods (19 ± 3 months) Normal training: 4 periods Intensive training: 1 period; (Load Δ: +200% time spent in anaerobic training zones) Cycling or triathlon; Maximal GXT, 10 s sprint, 30 s sprint, and TTE	Subjective complaints form (included questions related to disorders of sleep)	n/a	↓ Perceived sleep quality (within)
Woods et al. (2017)	17; 10 males and 7 females; 0/17 controls	Elite rowers (nominated for Australian Rowing Team selection during the study year); No $\overset{\cdot }{V}O_{2\text{max}}$ data	Uncontrolled before and after study; Pre-intensified: 7 days Intensified training: 28 days Post-intensified: 7 days (Load Δ: +21%, T2-minutes) Rowing; 5 km TT	Multicomponent training distress scale^d^ (provides a measure of sleep disturbances)	n/a	↔ Perceived sleep quality (within)
Woods et al. (2018)	14; 14 males and 0 females 0/14 controls	Trained cyclist with a consistent cycling history (>5 sessions/week, >10 h/week, >200 km week, >4 yrs) and regularly competing in grade A and B cycling races; 61.1 ± 6.2 ml.kg^-1^.min^-1^	Uncontrolled before and after study; Regular training: 28 days Increased training: 21 days Taper: 14 days; (Load Δ: +36%, Training Stress Score) Cycling; 15 s maximal sprints and 4000m maximal TT	Multicomponent training distress scale^d^ (provides a measure of sleep disturbances)	n/a	↔ Perceived sleep quality (within)

Abbreviations: GXT, graded exercise test; TT, time trial; TTE, time to exhaustion test; $\overset{\cdot }{V}O_{2\text{max}}$, maximal oxygen uptake; WBC, whole-body cryotherapy

^a^Control group was any group unexposed to an overreaching intervention (i.e., continued regular training)

^b^Included in the qualitative analysis only

^c^Compared to a control group or pre-overreaching

^d^Data included in the subjective meta-analysis

^e^Data included in the objective meta-analysis

## Results

### Search strategy

**Fig 1:**
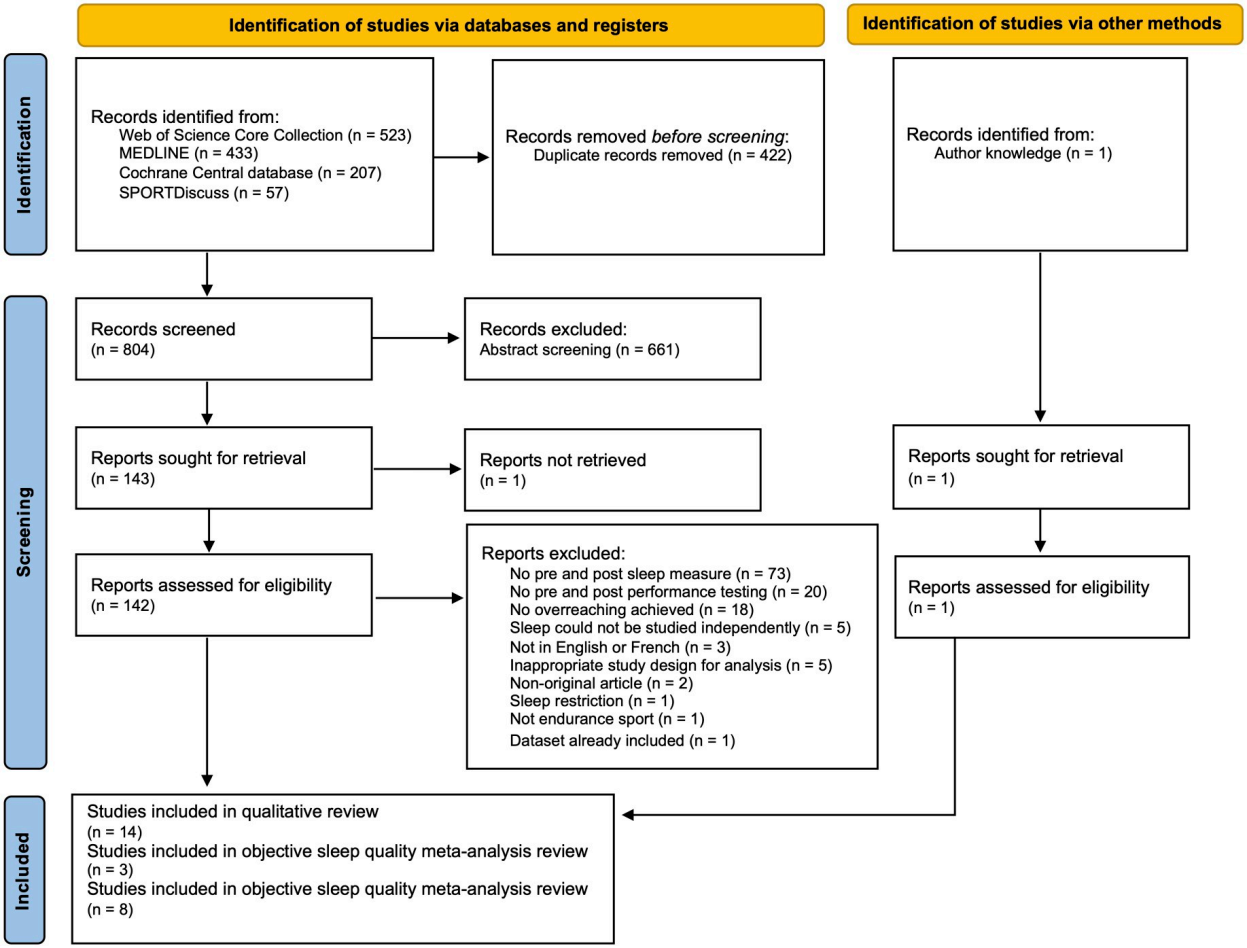
Preferred Reporting Items for Systematic Reviews and Meta-Analyses (PRISMA) flow diagram.

Following the database/grey areas search and the removal of duplicates, 805 articles were returned. Following the screening of article abstracts, 144 were eligible for examination via a full-text review. Fourteen were eligible for inclusion in the qualitative analysis [18–25, 31–36], reported in Table 1. Three articles were eligible for the meta-analysis examining the impact of overreaching on objective sleep quality [31, 35, 36] and eight were eligible for the meta-analysis examining the impact of overreaching on subjective sleep quality [18–22, 24, 25, 32–34]. Subgroup analyses were conducted for both meta-analyses to account for RCTs vs non-RCTs. A flow chart of the search strategy is presented in Fig 1.

### Design and measures

#### Study and participant characteristics

In the context of assessing the effect of overreaching on sleep quality, three studies were considered RCTs and eleven were considered non-RCTs. Of note, three studies implemented a crossover design but given that an overreaching protocol succeeded normal training during both crossover arms, these studies were considered non-RCTs for this review. Of the eligible studies, ten were of European and four were of Oceanic origin. The dates of publication ranged from 1992 to 2022. Across all studies, there was a total of 259 participants with a mean group age ranging from 19–35 yrs. Of these participants, 214 were included in subsequent analyses and 165 were exposed to an overreaching protocol and classified as overreached. Females were represented by 26 out of 259 (10%) participants. Based on the descriptions provided in the study texts, the training level of the participants ranged from endurance-trained to international-level athletes. Furthermore, no study that measured VO_2max_ data (number of studies [k] = 11, 79%) reported a subgroup mean lower than 52.8 ml.kg^-1^.min^-1^. The extracted data that comprise the qualitative analysis are summarised in Table 1.

#### Overreaching

The overreaching protocols were sixteen days in length, on average (ranging from 6–28 days). Of the fourteen studies, training hours during the overreaching intervention were reported for thirteen (93%), which equated to a mean value of 18.5 h/week (ranging from 7.3–27.8 h/week). The methods used to quantify differences in training load between the overreaching intervention and the control group (or baseline) were varied: total training h/week and/or time spent in anaerobic training zones (k = 5; 36%), sRPE x duration (k = 3, 21%), RPE x duration (k = 1, 7%), Training Stress Score (k = 1, 7%), and T2 minutes (k = 1, 7%). Three studies (21%) did not explicitly state how training load was quantified but it would be reasonable to assume they used total training h/week [20–22]. In terms of the exercise modalities implemented to incite overreaching, cycling (k = 5, 36%), rowing (k = 4, 29%), triathlon (k = 3, 21%), running (k = 2, 14%), and swimming (k = 1, 7%) were all utilised. One study (7%) allowed participants to use cycling or triathlon with the selection depending on the participant’s individual sport [25]. In order to detect overreaching, the following maximal performance tests were conducted using an individual or combined approach: time trials (k = 10, 71%), maximal incremental tests (k = 4, 29%), maximal sprint tests (≤ 30 s; k = 2, 14%), and time to exhaustion tests (k = 1, 7%). A minority of studies conducted ecologically valid performance tests (k = 4, 29%), while the majority included performance tests conducted within the research laboratory or using exercise machines (k = 13, 93%). Of the fourteen studies, twelve (86%) appeared to tailor the overreaching intervention to each participant’s baseline training (i.e. the relative change in training load was matched between participants but the absolute change may have differed), while two (14%) did not [18, 19].

#### Types of sleep measurements

Out of the fourteen studies included in this review, zero studies implemented polysomnography or a derivative such as electroencephalography to measure objective sleep quality. Actigraphy was utilised in three studies (21%) [31, 35, 36]. The manufacturer (CamNtech, Cambridge, England) of the actigraphy monitors was consistent across all three studies. Furthermore, sleep variables were collected every night of the study in the studies by Hausswirth et al. (2014) [31] and Schaal et al. (2015) [35]. In the study by Killer et al. (2015) [36], sleep variables were collected during all the baseline and intensified nights, but it is unclear if data was collected during the wash-out period. The actigraphs in all studies were worn on the wrist, however, the studies did not stipulate which arm the watch was fixed on (i.e., non-dominant vs dominant). The epoch lengths used were 30s [36], 1 min [31], and undefined [35]. All of the included studies used additional information to support the sleep-wake scoring algorithm (sleep log and/or time stamp button), yet the movement threshold set for the sleep analysis was undefined in all three.

Out of the fourteen studies included in the review, zero studies used a validated sleep quality questionnaire. Instead, subjective sleep quality was inferred from visual analogue scales or sleep quality questions embedded in sleep diaries and broader questionnaires. Thirteen of the included studies (93%) utilised some form of subjective measurement. Subjective sleep quality was inferred from: the RESTQ-Sport (k = 4, 29%) [21, 22, 24, 33], a 7-point sleep quality log (k = 3, 21%) [20, 31, 35], the Multicomponent Training Distress Scale (k = 2, 14%) [23, 24], the Karolinska Sleep Diary (k = 1, 7%) [32], a 10-point sleep quality log (k = 1, 7%) [34], a diary of sleep patterns (k = 1, 7%) [19], a 25-item overtraining questionnaire (k = 1, 7%) [18], and a subjective complaints form (k = 1, 7%) [25].

### Objective and subjective sleep outcomes

Of the three studies that measured objective sleep quality, only Hausswirth et al. (2014) [31] included a control group i.e. normal training. The remaining studies from Killer et al. (2015) [36] and Schaal et al. (2015) [35] implemented crossover designs. Nonetheless, given that an overreaching protocol was implemented during both crossover arms, only baseline and post-intervention sleep data were considered for this review (to mimic a before and after trial design). A range of objective sleep variables was reported across all three studies including bedtime, getup time, time in bed, total sleep time, sleep-onset latency, sleep efficiency, percentage sleep time, number of wake bouts, sleep fragmentation, immobile minutes, and %move time. Nonetheless, only relevant objective sleep continuity variables were extracted and reported in Table 1.

Of the thirteen studies that measured subjective sleep quality, only three included a control group i.e. normal training [31–33]. Achten et al. (2004) [19] implemented a crossover design with an overreaching protocol during both arms and, as a result, was reviewed as a before and after trial for this review. The remaining eleven studies were before and after trials. Of the 5 studies excluded in the meta-analysis (reasons provided in “*Subjective sleep quality*” section), sleep quality from the overreaching intervention was reported as decreasing by three [18, 22, 25] and unchanged by two [19, 20]. All subjective sleep quality findings across each study are displayed in Table 1.

### Meta-analyses

#### Objective sleep quality

**Fig 2:**
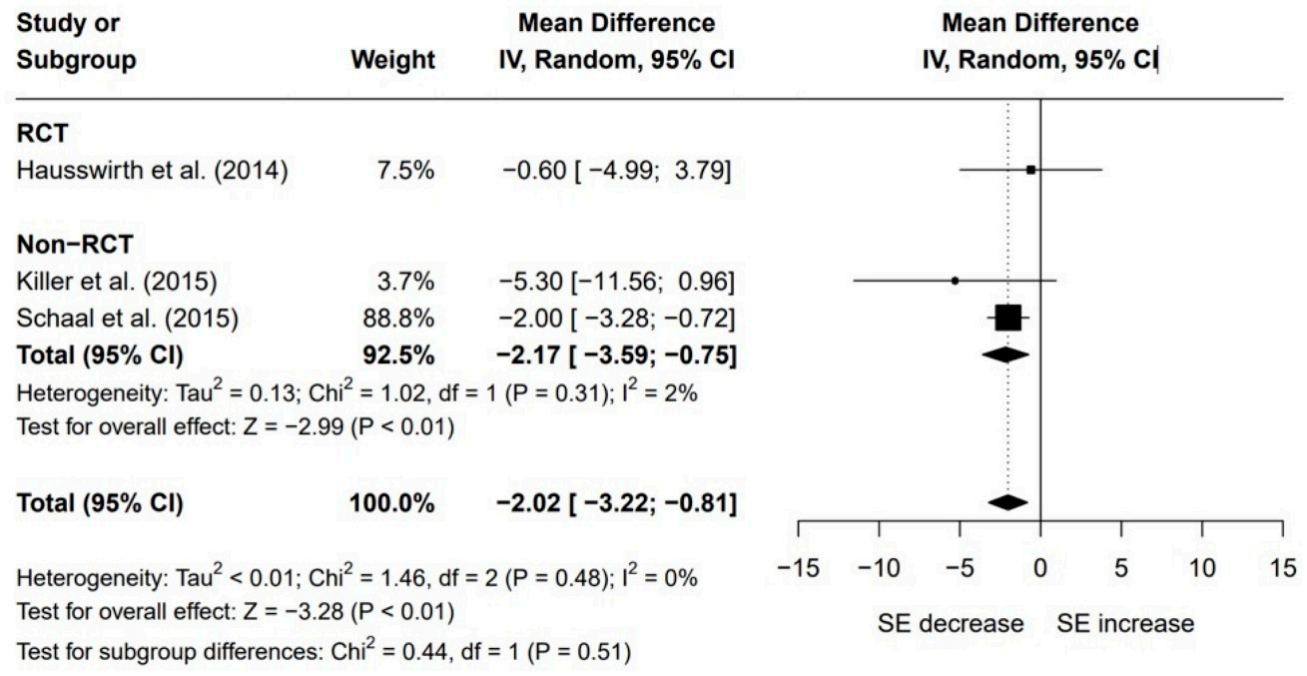
Objective sleep quality plot. Data are presented as mean differences and 95% confidence intervals (CIs). Effects to the left of the solid line (0) indicate a decrease in sleep efficiency (SE), whereas effects to the right of the solid line indicate an increase in sleep efficiency with overreaching. The dotted line indicates the effect line of included studies within this meta-analysis. Random effects restricted maximum likelihood method used.

For the three studies that objectively measured sleep, sleep efficiency was the only sleep continuity variable reported with raw data (mean, standard deviation, sample size and was included in the meta-analysis, see Fig 2. When comparing objective sleep quality following the overreaching intervention to baseline (or a control), there was a significant reduction in sleep efficiency (mean difference = -2.0%; 95% CI -3.2, -0.8%; Glass’ Δ = -0.83; p < 0.01). No significant heterogeneity was found (Q = 1.46, df = 2, p = 0.48; I^2^ = 0%). A subgroup analysis highlighted that non-RCTs had a large significant effect on the overall analysis (mean difference = 2.2%; 95% CI -3.6, -0.8; Glass’ Δ = -1.23; p < 0.01) and displayed low heterogeneity (Q = 1.02, df = 1, p = 0.31; I^2^ = 2%). Due to insufficient sample size (k = 1), subgroup outputs for RCTs were not producible.

#### Subjective sleep quality

**Fig 3:**
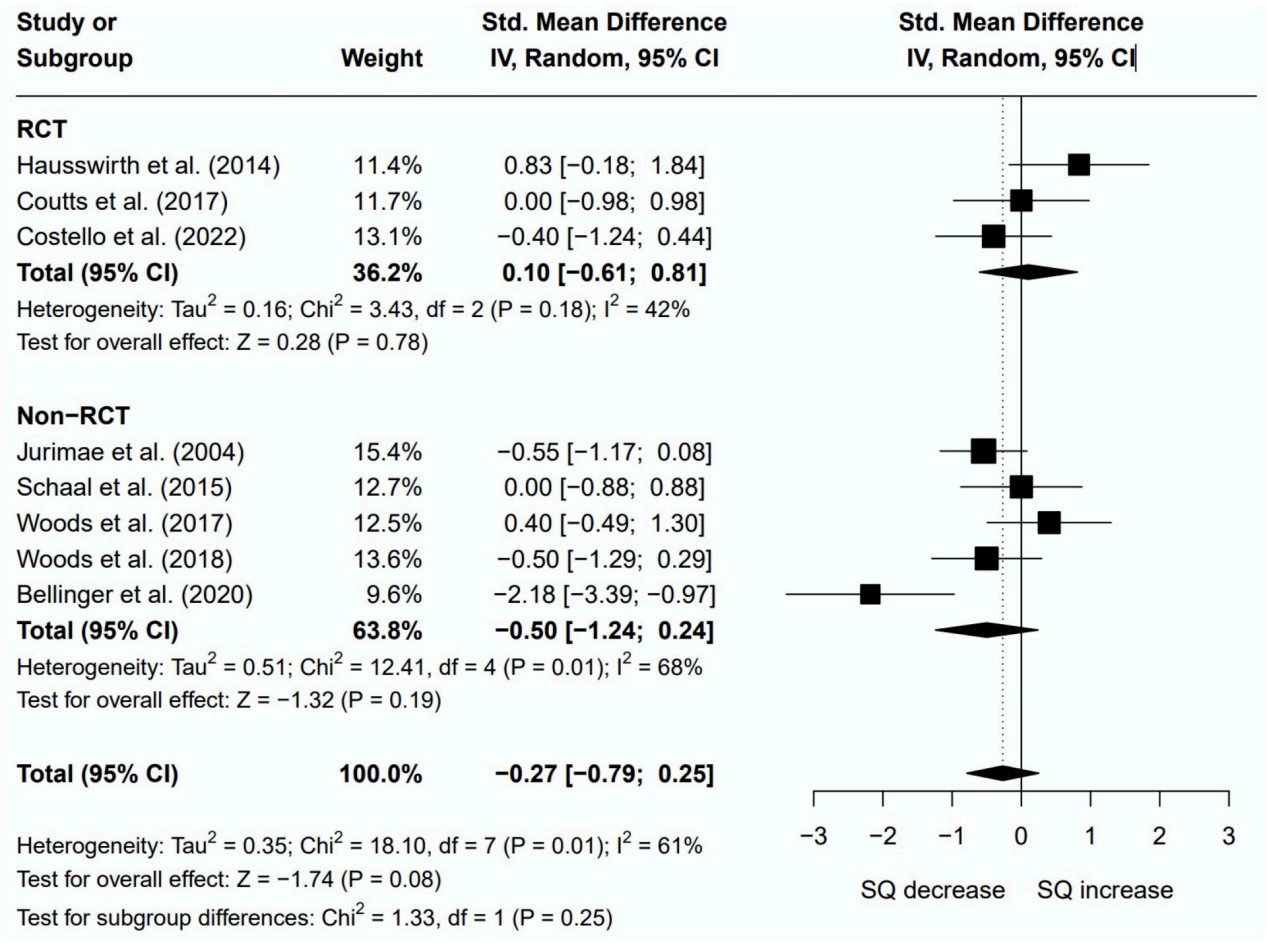
Subjective sleep quality plot. Data are presented as Glass’ Δ and 95% confidence intervals (CIs). Effects to the left of the solid line (0) indicate a decrease in subjective sleep quality (SQ), whereas effects to the right of the solid line indicate an increase in subjective sleep quality with overreaching. The dotted line indicates the effect line of included studies within this meta-analysis. Random effects restricted maximum likelihood method used.

Eight of the studies that subjectively measured sleep were included in the meta-analysis, see Fig 3. Five studies were excluded for the following reasons: no raw data, only p-value reported (k = 2) [18, 22], binary data (k = 1) [25], no data, only a statement of the results reported (k = 1) [19], and authors concern over duplicating data within the meta-analysis (k = 1) [20]. The authors identified similarities between three studies from the same research group [20–22]. The research group was contacted for clarification on the datasets used, but no response was received. Therefore, while all studies are included in the qualitative analysis, only one study [21] was included in the meta-analysis. When comparing subjective sleep quality following the overreaching intervention to baseline (or a control), there was no effect of overreaching on subjective sleep quality (Glass’ Δ = -0.27; 95% CI -0.79, 0.25; p = 0.08) but significant heterogeneity (Q = 18.10, df = 7, p = 0.01; I^2^ = 61%). A subgroup analysis highlighted that RCTs and non-RCTs displayed a non-significant effect when analysed independently (Glass’ Δ = 0.10; 95% CI -0.61, 0.81; p = 0.78 and Glass’ Δ = -0.50; 95% CI -1.24, 0.24; p = 0.19, respectively). Heterogeneity was not present for RCTs (Q = 3.48, df = 2, p = 0.18; I2 = 42%), but highly significant for non-RCTs (Q = 12.41, df = 4, p = 0.01; I2 = 68%).

### Risk of bias

**Fig 4:**
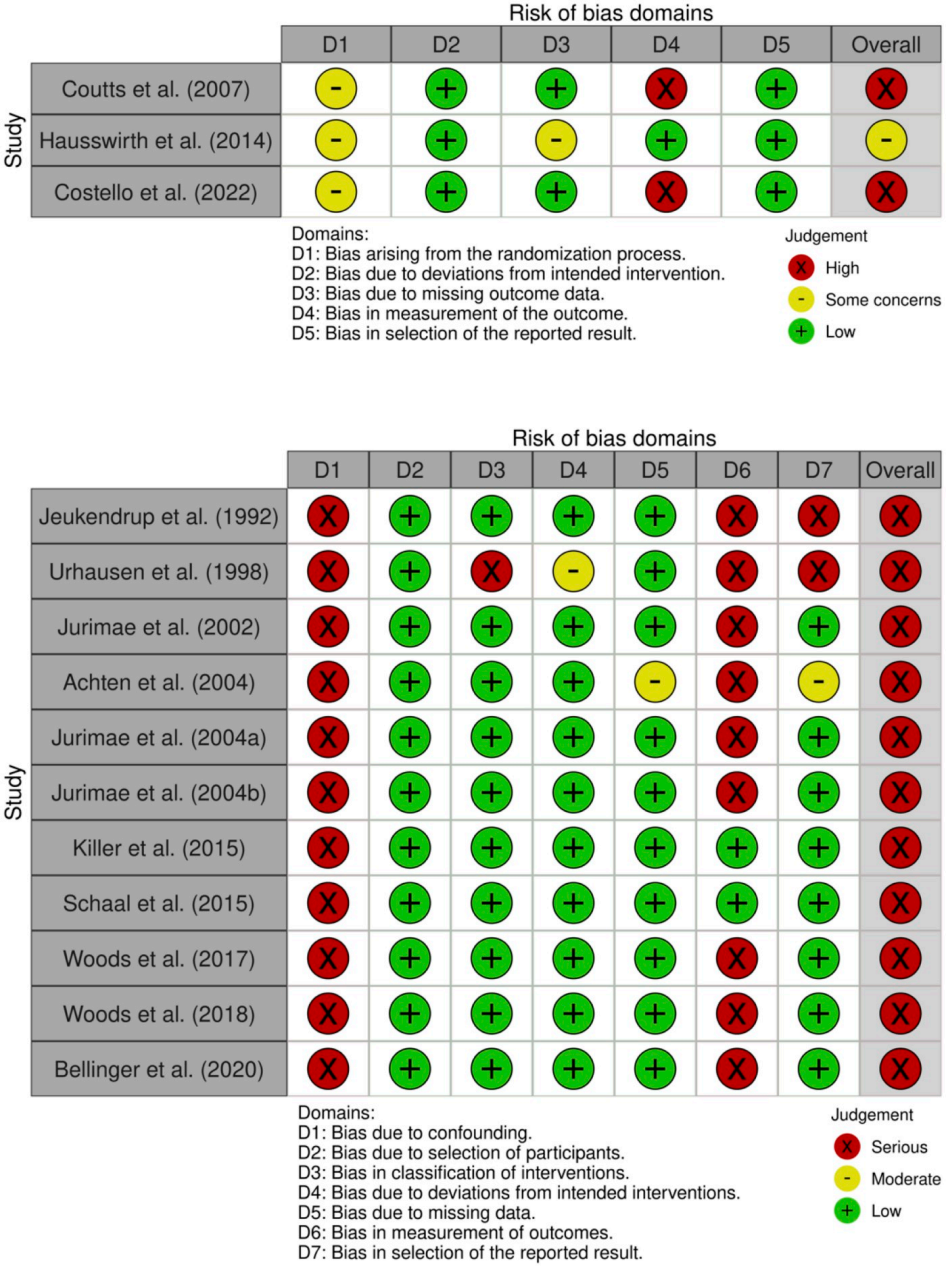
Risk of bias assessment for RCTs (upper) and non-RCTS (lower).

The risk of bias assessment for the RCTs and non-RCTS is highlighted in Fig 4. The main area of shared concern for RCTs and non-RCTs was "bias in measurement of outcomes”. This is the consequence of only 21% of the studies using objective measures of sleep quality. Subjective measures, particularly in sleep research, can be biased by the assigned intervention. Another area of some concern, in the context of RCTs, was “bias arising in the randomisation process”. While each study stated their sample was randomised, none presented appropriate detail of the allocation sequence and whether this process was concealed from the study personnel. Similarly, all non-RCTs studies presented serious concern regarding “bias due to confounding”. This judgement was made due to the pre- vs post-overreaching comparisons used to infer changes in sleep quality. No study collected an appropriate quantity of pre- and post-measurements combined with an analysis method to account for time as a confounding variable. As a consequence, the study designs were considered as “before and after trials” instead of the more appropriate “interrupted time series trials”.

## Discussion

The aim of this systematic review was to describe the impact of overreaching on sleep outcomes within the endurance research field and determine the effect of endurance-based overreaching on sleep quality. The main findings were that overreaching had a large effect on objective sleep quality (sleep efficiency), albeit a small change, and no effect on subjective sleep quality (perceived sleep quality). Furthermore, subgroup analyses accounting for the effects of RCTs compared to non-RCTs did not alter the outcomes of the review. Thus, overreaching from endurance-based training has the potential to disrupt sleep quality.

Sufficient training load is essential for the adaptations that are necessary to improve the exercise capacity of athletes. However, increased training load and overreaching are often believed to pose a threat to sleep [12, 13], an important component of the recovery process. While it is commonly acknowledged that overreaching can negatively impact sleep quality, a comprehensive review of the available literature did not exist before this systematic review and meta-analysis. This study adds context for athletes by summarising our current knowledge on the impact of overreaching on sleep in endurance sports and for researchers by highlighting the current state of the field and the future work that is required.

### Design and measures

A positive aspect of the included studies was in relation to the performance level of the participants. Considering De Pauw et al. (2013) [37] and Decroix et al. (2016) [38] athlete classification, of the studies that reported VO_2max_ data, all but one [33], recruited participants that were either trained, well-trained or professional athletes (VO_2max_: 55–64.9, 65–72, > 72 ml.kg^-1^.min^-1^, respectively). As this classification system was developed for cycling-based research, it should be used cautiously but approximates the level of endurance-trained participants in these studies. Due to the intensive training required to conduct an overreaching study, there is an increased possibility of dropouts, therefore, recruiting trained participants with extensive experience and familiarisation with the exercise modality is critical. Another important aspect relates to the duration of the overreaching period. While overreaching does not have a duration criterion attached to it, it is reasonable to assume that shorter durations may instead reflect acute fatigue. Acute fatigue could be considered a short-term response to recent training which contrasts with overreaching which may persist for up to one to three weeks [3]. The average duration of the overreaching interventions in this review was sixteen days which would appear to be a sensible balance between enticing overreaching and mitigating the risks of dropouts due to injury, illness, or motivation. Similarly, a sufficient number of days is required to capture changes in sleep quality. Sleep displays high inter-daily variability, and as a consequence, at least one week of data is often recommended [39]. Furthermore, the timing of changes in sleep quality along the overreaching spectrum is unknown and may be missed if insufficient days are recorded after overreaching occurs. As a consequence, ten days of an overreaching protocol should be considered the minimum requirement when the primary outcome is sleep to help ensure multiple days of sleep measurements can be collected and an adequate number of days reflect an overreached state.

### Meta-analyses

#### Objective sleep quality

The results from the objective meta-analysis showed that overreaching from endurance training caused a significant reduction in actigraphic sleep efficiency. This finding supports the belief that overreaching is associated with sleep disturbances garnered from anecdotal evidence in the wider sports community. In addition, literature reviews from over twenty years ago suggested sleep may be disturbed by overtraining [2] while a recent consensus statement acknowledged this possibility too [14]. Nonetheless, an important consideration is whether the changes in objective sleep quality found in this review are clinically meaningful. While all three studies trended towards decrements in sleep efficiency from overreaching, the overall mean difference between sleep quality in an overreached state compared to a non-overreached state was only 2%. Whether this change is clinically significant is dubious. For example, mean scores for sleep efficiency in the overreached state did not enter a range generally agreed upon as an indicator of poor sleep quality (< 75%) [16]. Nonetheless, despite such small changes it is wise to acknowledge this effect as the possibility that further decrements would ensue with the continuation of overreaching and eventual overtraining cannot be discounted.

In order to strengthen the analysis, it would have been beneficial to have conducted meta-analyses with the other objective variables that indicate poor sleep quality. However, sleep efficiency was the only variable with raw data in each of the three studies using objective measures. Importantly, sleep efficiency can be considered a global measure which includes sleep-onset latency, wake after sleep onset and the number of awakenings in its output. Therefore, these variables would have only acted as an extension to sleep efficiency by identifying the wake times location relative to the sleep period.

### Subjective sleep quality

In contrast to objective sleep quality, the results from the subjective meta-analysis showed that endurance-based overreaching did not alter perceived sleep quality. Only two studies measured both objective and subjective sleep outcomes, however, this discrepancy was also found in both [31, 35]. Although it is unclear why this discrepancy occurred, there are several plausible explanations: 1) the change in objective sleep was too small for the individual to be consciously aware, 2) the changes in actigraphic sleep quality may suggest increased movement that does not transpire to poorer sleep quality from an electrophysiological perspective, or 3) the combination of different subjective sleep quality scales in the meta-analysis reduced sensitivity. As previously eluded to, the changes in objective sleep quality from overreaching may not be clinically relevant, thus, the effect shows no significant impact on the participant’s perception. Regretfully, more studies using both objective and subjective data could have helped investigate this hypothesis further. In order to determine whether changes in actigraphic sleep quality may be related to increased movement, electroencephalography measures would be required to confirm that the increased movement is not increasing the number of epochs scored as wake or altering sleep stages. Lastly, while it is possible using a combination of different scales could have reduced the sensitivity of the meta-analysis, this is unlikely, as each scale measured similar outcomes that could be broadly interpreted as perceived sleep quality. Instead, a more plausible explanation is that each individual scale may lack validity or sensitivity to assess changes in sleep quality.

### Risk of bias

Another very important finding from this systematic review was the high risk of bias and poor quality of studies conducted in this area. All but one study [31] was judged as having an overall high risk of bias. A primary reason for this was that 79% of studies utilised only subjective measures of sleep which are subject to bias in an overreaching intervention due to an inability to conceal the intervention from the participants. As a consequence, there is a high risk of the subjective outcome being influenced by knowledge of the intervention received. Regarding the RCTs within this review [31–33], none of them provided information regarding the randomisation of the allocation sequence or concealment of this process. Confounding bias was also a problem in the non-RCTs as none of the studies used an appropriate design or analysis method to account for time-varying confounders [18–25, 34–36]. Therefore, without a control group, whether changes are intervention- or time-related cannot be discerned.

Apart from bias, the quality of studies is also of concern. Only three studies were RCTs designed for the purpose of assessing the effect of overreaching on sleep outcomes. For example, while the studies of Killer et al. (2015) [36] and Schaal et al. (2015) [35] were comprehensive crossover interventions, they were not appropriate for solely investigating the effect of overreaching on sleep outcomes, rather the ability of recovery interventions to mitigate overreaching. Therefore, the effect of overreaching on sleep outcomes was a secondary consideration like many other studies in this review. Unfortunately, no studies utilised polysomnography which is considered the gold standard for measuring sleep and only three studies used any objective measure [31, 35, 36]. Actigraphy was the sole objective measure used, however, caution must be taken when interpreting actigraphic data as the scoring of sleep and wake is underpinned by movement data rather than true physiological signals. Furthermore, as mentioned previously, the subjective instruments used to measure sleep have not been validated to measure sleep quality. Valid questionnaires to identify a range of sleep disorders do exist which may be more appropriate for future research [40].

### Limitations

This systematic review and meta-analysis is not without limitations. In particular, the high risk of bias across the eligible studies restricts confidence in the results. While certain features are difficult to control in this type of research, such as blinding participants to an overreaching intervention and balancing the risk of injury and illness with sufficient training stress, other areas could be improved. As discussed in the *“Risk of bias”* section, this includes but is not limited to the use of a control group, using gold-standard and objective methods, and designing a study with the primary purpose of investigating the effect of overreaching on sleep outcomes. Only three studies were eligible for inclusion in the objective meta-analysis, given the small samples and effects of each, additional studies may have helped support the results. In contrast, while a sufficient number of studies were included in the subjective meta-analysis, there was significant heterogeneity between them due to the use of different scales across studies. However, each measured outcome could be broadly interpreted as perceived sleep quality which supports our approach of combining these scales. Given the inclusion and exclusion criteria of this review, many overreaching articles were excluded. For example, studies that assessed outcomes after overreaching already occurred, studies that did not appropriately specify the timeline of measurements and overreaching windows, or studies that did not confirm overreaching with statistically significant performance decrements, were all excluded. While this may have resulted in the loss of potentially valuable articles, it also prevented the inclusion of articles that were misleading and difficult to interpret with regard to our research question. While we only included studies that reported a decrement in performance after the overreaching intervention, there was heterogeneity in the performance tests used. Likely, some performance tests lack sensitivity to capture overreaching, while others may overstate the overreached state. However, currently it is not clear which performance tests are most appropriate for this purpose, thus, we did not limit our selection to any in particular. Similarly, some eligible studies had short overreaching intervention periods. Short intervention periods may present problems as the state transition to overreaching may develop continuously over the intervention period. Therefore, if researchers are averaging values across the intervention period this may also include nights when the athletes have yet to reach an overreached state. Also, while the final nights may reflect overtraining, the limited number of nights measured in this state may lack sensitivity due to the nightly variability that occurs naturally with sleep. Nonetheless, the literature does not indicate a time duration that is associated with the development of overreaching, so it would be challenging to exclude studies based on a short intervention period. Instead, we encourage researchers to implement a logical intervention period in future studies. Of course, this systematic review and meta-analysis was restricted to endurance-based sports which limits the generalizability of the results. Nonetheless, as discussed in the introduction this approach was important to dissociate the effects of overreaching on sleep quality from other sports-specific confounding factors. Finally, the search strategy was limited to English and French articles. Therefore, it is possible that we missed studies in other languages.

### Practical applications

From a practical perspective, while sleep quality may be sensitive to overreaching, based on the current evidence, sleep tools may be difficult to implement as markers of overreaching. Subjective tools do not appear to be sensitive enough to detect overreaching, and while objective markers may show promise, the small changes may be hard to detect in a cohort of athletes. While we acknowledge sleep changes may become more noticeable when functional overreaching transitions to non-functional or overtraining, given the diversity in research and small nuances between these state transitions, such states were not separated in this analysis. Furthermore, it is plausible that when sleep impairments do occur, the athlete has already reached a detrimental position in the context of training optimisation. Therefore, we would advise those working with athletes to avoid solely depending on sleep measures to indicate overreaching but instead use it with other markers not limited to perception of training effort, mood, and soreness.

### Future directions

Given the results of this systematic review and meta-analysis, and subsequent discussion, it is evident that more research is required in this area. In particular, research studies that are designed specifically to address the question of whether overreaching affects sleep are necessary. Thus, the following points are to support future research in this area.

- Implement an appropriate study design, such as an RCT, with sleep quality being the primary outcome of interest. If a non-RCT is used, ensure enough data points are collected pre- and post-intervention to account for time-varying confounders (interrupted time series trial) or participants crossover between an overreaching and non-overreaching arm (crossover trial). An appropriate baseline or familiarisation period and a post-intervention taper period should also be implemented (≥ one week).
- Use both objective and subjective measures to capture changes in sleep quality. Ideally, utilising electroencephalography which could help determine whether increased movement impairs sleep quality. If actigraphy is used, be careful to include the set-up and analysis settings e.g., movement threshold. Furthermore, validated instruments should be considered to assess subjective sleep [40].
- Implement an overreaching period of sufficient length to ensure that sleep quality is being assessed when the athlete has reached an overreached state. A minimum of ten days is recommended, however, to avoid injury/illness of the athletes the total change in training load must be cautiously monitored.
- Individual pre- and post-results should be reported for performance testing to display the extent of overreaching or lack of in certain individuals.
- Implement an appropriate training schedule with respect to the sleep-wake patterns of the participants and report bed- and wake times across each phase of the study period. Many of the included studies did not stipulate when the training sessions were conducted and whether this could have altered the participants’ regular sleep-wake profile. As an example, if additional or longer sessions were added in the morning this may have caused the participants to wake prior to their habitual waketime. Furthermore, an attempt should be made to keep the athletes sleeping in their natural environment. Otherwise, a consistent environment should be implemented across each phase of the study to avoid changes in the sleep environment acting as a confounder to sleep quality.
- Finally, future studies should include effect sizes and discuss whether the magnitude of changes are clinically meaningful.

## Conclusion

While acknowledging the need for more high-quality studies in this area, it appears that overreaching from endurance-based training can lead to small deteriorations in objective sleep quality. In contrast, these effects do not appear to significantly influence the individual’s perception of sleep quality. However, due to the high risk of bias and poor quality of studies conducted in this area, we encourage researchers to continue investigating this area while being considerate of previous limitations.

## Supporting information

S1 Appendix Search strategy. (DOCX)

S2 Appendix PRISMA checklist. (PDF)
