# Supplementary material for: Does overreaching from endurance-based training impair sleep: A systematic review and meta-analysis
Source: PLoS One. 2024 May 29;19(5):e0303748. doi: 10.1371/journal.pone.0303748 (PMC11135706; doi:10.1371/journal.pone.0303748)
Supplement: S1 Appendix — (DOCX) [file pone.0303748.s001.docx]

**Web of Science Core Collection:**

(((ALL="Sleep") OR (ALL="mood") OR (ALL="rest") OR (ALL="insomnia") OR (ALL="recovery")) AND ((ALL="Overreaching") OR (ALL="Overtraining") OR (ALL="maladaptation") OR (ALL="burnout") OR (ALL="underperformance syndrome") OR (ALL="underrecovery") OR (ALL="increased training") OR (ALL="intensified training")) AND ((ALL="endurance") OR (ALL="aerobic") OR (ALL="interval") OR (ALL="running") OR (ALL="cycling") OR (ALL="swimming") OR (ALL="triathlon") OR (ALL="canoeing") OR (ALL="skiing") OR (ALL="rowing") OR (ALL="kayaking") OR (ALL="marathon") OR (ALL="Ironman") OR (ALL="ultra")))

**MEDLINE;**

("Sleep"[All Fields] OR "mood"[All Fields] OR "rest"[All Fields] OR "insomnia"[All Fields] OR "recovery" [All Fields]) AND ("Overreaching"[All Fields] OR "Overtraining"[All Fields] OR "maladaptation"[All Fields] OR "burnout"[All Fields] OR "underperformance syndrome"[All Fields] OR "underrecovery"[All Fields] OR "increased training"[All Fields] OR "intensified training"[All Fields]) AND ("endurance"[All Fields] OR "aerobic"[All Fields] OR "interval"[All Fields] OR "running"[All Fields] OR "cycling"[All Fields] OR "swimming"[All Fields] OR "triathlon"[All Fields] OR "canoeing"[All Fields] OR "skiing"[All Fields] OR "rowing"[All Fields] OR "kayaking"[All Fields] OR "marathon"[All Fields] OR "Ironman"[All Fields] OR "ultra"[All Fields])

**Cochrane Central Database:**

1. MeSH descriptor: [Sleep] explode all trees

2. ("sleep") OR ("mood") OR ("rest") OR ("insomnia") OR ("recovery")

3. #1 OR #2

4. MeSH descriptor: [Overtraining Syndrome] explode all trees

5. ("Overtraining") OR ("Overreaching") OR ("maladaptation") OR ("burnout") OR (underperformance syndrome)

6. ("underrecovery") OR ("increased training") OR ("intensified training")

7. #5 OR #6

8. MeSH descriptor: [Endurance Training] explode all trees

9. ("endurance") OR ("aerobic") OR ("interval") OR ("running") OR ("cycling")

10. ("swimming") OR ("triathlon") OR ("canoeing") OR ("skiing") OR ("rowing")

11. ("kayaking") OR ("marathon") OR ("Ironman") OR ("ultra")

12. #8 OR #9 OR #10 OR #11

13. #3 AND #7 AND #12

**SPORTDiscus:**

Sleep AND ("Overreaching" OR "Overtraining" OR "maladaptation" OR "burnout" OR "underperformance syndrome" OR "underrecovery" OR "increased training" OR "intensified training") AND ("endurance" OR "aerobic" OR "interval" OR "running" OR "cycling" OR "swimming" OR "triathlon" OR "canoeing" OR "skiing" OR "rowing" OR "kayaking" OR "marathon" OR "Ironman" OR "ultra")
